# Seeing the “Big” Picture: Big Data Methods for Exploring Relationships Between Usage, Language, and Outcome in Internet Intervention Data

**DOI:** 10.2196/jmir.5725

**Published:** 2016-08-31

**Authors:** Jordan Carpenter, Patrick Crutchley, Ran D Zilca, H Andrew Schwartz, Laura K Smith, Angela M Cobb, Acacia C Parks

**Affiliations:** ^1^ Positive Psychology Center University of Pennsylvania Philadelphia, PA United States; ^2^ Penn Medicine Social Media and Health Innovation Lab Penn Medicine Center for Health Care Innovation University of Pennsylvania Philadelphia, PA United States; ^3^ Happify New York, NY United States; ^4^ Computer Science Stony Brook University Stony Brook, NY United States; ^5^ Department of Psychology Hiram College Hiram, OH United States

**Keywords:** well-being intervention, big data, qualitative analysis, linguistic analysis, word cloud, multilevel modeling

## Abstract

**Background:**

Assessing the efficacy of Internet interventions that are already in the market introduces both challenges and opportunities. While vast, often unprecedented amounts of data may be available (hundreds of thousands, and sometimes millions of participants with high dimensions of assessed variables), the data are observational in nature, are partly unstructured (eg, free text, images, sensor data), do not include a natural control group to be used for comparison, and typically exhibit high attrition rates. New approaches are therefore needed to use these existing data and derive new insights that can augment traditional smaller-group randomized controlled trials.

**Objective:**

Our objective was to demonstrate how emerging big data approaches can help explore questions about the effectiveness and process of an Internet well-being intervention.

**Methods:**

We drew data from the user base of a well-being website and app called Happify. To explore effectiveness, multilevel models focusing on within-person variation explored whether greater usage predicted higher well-being in a sample of 152,747 users. In addition, to explore the underlying processes that accompany improvement, we analyzed language for 10,818 users who had a sufficient volume of free-text response and timespan of platform usage. A topic model constructed from this free text provided language-based correlates of individual user improvement in outcome measures, providing insights into the beneficial underlying processes experienced by users.

**Results:**

On a measure of positive emotion, the average user improved 1.38 points per week (SE 0.01, t122,455=113.60, *P*<.001, 95% CI 1.36–1.41), about a 27% increase over 8 weeks. Within a given individual user, more usage predicted more positive emotion and less usage predicted less positive emotion (estimate 0.09, SE 0.01, t6047=9.15, *P*=.001, 95% CI .07–.12). This estimate predicted that a given user would report positive emotion 1.26 points higher after a 2-week period when they used Happify daily than during a week when they didn’t use it at all. Among highly engaged users, 200 automatically clustered topics showed a significant (corrected *P*<.001) effect on change in well-being over time, illustrating which topics may be more beneficial than others when engaging with the interventions. In particular, topics that are related to addressing negative thoughts and feelings were correlated with improvement over time.

**Conclusions:**

Using observational analyses on naturalistic big data, we can explore the relationship between usage and well-being among people using an Internet well-being intervention and provide new insights into the underlying mechanisms that accompany it. By leveraging big data to power these new types of analyses, we can explore the workings of an intervention from new angles, and harness the insights that surface to feed back into the intervention and improve it further in the future.

## Introduction

As Internet interventions become increasingly popular—in research settings, but even more so in industry—they yield large datasets that can be used for research purposes. These datasets often fall under the umbrella of “big data,” where big data is defined as a dataset so large and complex that traditional data analytic approaches cannot easily handle them [1]. While these datasets are often not designed upfront to answer research questions, and therefore often do not have control groups, they are large in size, are rich in content, and offer a view into the intervention that allows users to interact with an intervention naturalistically. Big intervention data, therefore, offer the opportunity to test interventions in the real world, where interventions are actually being found by individuals and used naturalistically. Moreover, since the data are produced during normal usage, they can be immediately useful as a means for assessing not only whether the intervention is effective, but also which aspects and parts of it are more effective than others and how the intervention can be modified to become more effective. Such iterative processes take advantage of the short implementation cycles of software as opposed to more traditional medical interventions.

Specialized analytic methods are necessary to handle the amount and frequency of data given for a particular user and to accommodate a very large number of users. Despite the extra care needed to analyze big data, there are some substantial benefits to using a big data approach. First, whereas more traditional intervention evaluation studies are usually constrained by budgetary concerns (each participant costs money in order to encourage retention), openly available products generally aim to acquire as many users as possible. The resulting potential sample size is massive and grants the power to do a class of analyses that could not be considered in a typical study with 100–200 (or even 500–600) users per cell. The large number of participants also provides enough power for moderator analyses, which is a capability often available only to meta-analyses. Second, the sample derived from an existing intervention potentially has much greater external validity because of the lack of a highly controlled experimental setting. Third, by analyzing data outside of the restrictions of a traditional randomized, controlled design (including unstructured data such as text produced by users), researchers are opened up to a new variety of potential research questions [2] and can directly examine the relationship between spontaneous usage and outcome.

Of course, the analysis of naturalistic big data has its own problems, especially in an industry context. Researchers often have less flexibility in what they can ask participants, because excessive in-product assessment can reduce retention [1]. researchers are therefore limited to knowing only what participants tell them over the course of naturally using the product. Perhaps most problematically, there is no formal control group. It is easy, therefore, to relegate any data analysis that springs from a real-world Internet intervention to a lower status than that from a randomized controlled trial (RCT) [3]. However, some of these issues are addressable with the right kind of data.

Although there have been write-ups of psychological interventions that explore efficacy or effectiveness without a control group, these are largely treated as uncontrolled pilot studies [4] where no special approach was taken to offer an alternative to the control group. One common study design in this context, the cohort study, is essentially a longitudinal study in which a sample is followed and tracked over time. Watching outcomes unfold naturally in this way allows researchers to establish temporal precedence. The hypothesized cause comes before the hypothesized effect, which is superior to a cross-sectional study when establishing causality [5]. This type of design can often yield a more externally valid sample, with fewer screening criteria or other restrictions to sample membership, which makes generalization more appropriate. However, cohort studies are vulnerable to problems with bias, because the lack of random assignment may mean systematic differences between those who do well and those who do not. As such, there is a substantial gap in the literature regarding rigorous approaches to testing interventions in uncontrolled settings [2]. There are not yet best practices for doing so.

One possible alternative approach stems from the self-controlled case series approach, most commonly used in medicine [6,7]. Like in a cohort study, participants are tracked over time in a self-controlled case series study, but the emphasis is not on averaging individuals together or on making comparisons between individuals. Instead, the emphasis is on within-person changes on (and potentially interactions between) observed variables [6]. In this study, we applied some of the strengths of a cohort study with a statistical approach that might be used in a self-controlled case series study (study 1). Specifically, we used ongoing data about user engagement available through a website and app. Our objective was to conceptualize usage not as a trait or static variable (eg, user 1 was high usage, user 2 was low usage) but as a dynamic, constantly changing variable that may be tied to higher or lower outcomes as it varies. Whereas many RCTs aim to standardize or maximize engagement (ideally, every participant would show 100% engagement) [8], multilevel models can harness that variation, creating a dose-response relationship between behavior and outcome.

An additional goal was to explore an exciting aspect of big data: its potential to fuel linguistic analysis of the text produced by users and entered into a site (study 2). Large amounts of language can be mined to automatically reveal latent psychological processes [9,10]. Such analyses can permit us to examine not only effectiveness, but also processes in ways that would not be possible without large volumes of text. Commercial platforms can allow natural language data to accrue daily, and the text can be mined for broad-scale patterns in language that reflect a person’s process of improvement. In this way, our work is an example of an existing literature that uses passive forms of data collection, such as search behavior and phone sensors [11], to measure and characterize psychological constructs and to develop opportunities for targeted intervention [12].

To summarize, the goal of our study was to broach several interesting research questions that become possible to ask when working with a massive, real-world intervention dataset. We began in study 1 by using multilevel modeling to establish the overall effects of a Web-based self-help platform on well-being, tracking the improvement over time as it varied within an individual [5,13]. By focusing on within-person variation, it was possible to address a common problem in uncontrolled data analysis, that is, systematic biases between different users who exhibit generally high or generally low usage, by emphasizing how each person’s improvement varied based on his or her usage. The question shifted from “Did users who get the intervention improve?” to “Did users feel better during weeks that they used the intervention more and worse during weeks that they used it less?”

In line with previous work finding that Internet interventions are able to improve well-being effectively, we expected that users would exhibit higher well-being during periods in which they used the intervention more frequently [14,15]. Consistent with previous work on the relationship between effort and outcomes, within any given user, more usage would be correlated with higher well-being [16]. Furthermore, consistent with moderator analyses from meta-analyses of behavioral well-being interventions, we expected that this effect would be magnified for users who began with lower well-being [17,18].

In study 2, we then used linguistic analysis to gain a descriptive picture of this effect, that is, to visualize what people were saying (and, we might extrapolate, what psychological processes they were experiencing) that might help explain why they experienced improvements. While our overall hypothesis was that certain patterns of word usage would be related to well-being, we did not have specific predictions about which words might be most strongly associated.

## Methods

We drew all data from the user base of Happify, a Web-based platform that offers techniques grounded in positive psychology, cognitive behavioral therapy, and mindfulness. Happify can be used on the Internet, via an app (Android and iOS), or both. People find Happify through media coverage, word of mouth, social media, and paid advertising across the Internet.

Consistent with previous research using commercial well-being apps [19], participants gave semipassive consent by accepting a user agreement that explained that their data may be used for research. Specifically, the terms and conditions of Happify stated that “Information that we collect about you also may be combined by us with other information available to us through third parties for research and measurement purposes, including measuring the effectiveness of content, advertising, or programs. This information from other sources may include age, gender, demographic, geographic, personal interests, product purchase activity or other information.”

We assessed 3 demographic questions—age, employment status, and sex—in all users. A fourth question about number of children was added later, and therefore only asked of a subset of users. The dataset contained data from users whose accounts were created between December 1, 2014 and May 1, 2016, and the 8-week intervention period began at the time they completed their first assessment.

### Content of Happify

We organized activities into the following 5 categories using the acronym STAGE: savor (mindfulness activities) [20,21], thank (gratitude activities) [22,23], aspire (optimism, best possible selves, goal setting, and meaning or purpose activities) [24,25], give (kindness, prosocial spending, and forgiveness activities) [26], and empathize (self-compassion and perspective-taking activities) [27]. Not all users used the same activities, as there were many possible ways to progress through the site. Users selected from many possible tracks, which were collections of activities that targeted a particular goal or problem, such as coping better with stress or improving one’s romantic relationship. Any given track drew activities from several areas of STAGE, and the track recommended activities to the user on a daily basis that were customized to his or her particular goal. Users also received automated reminder emails and mobile phone notifications (if they used the app). However, users were not necessarily constrained by a track, either; some chose to also use a free play section, where they could pick and choose individual activities as they liked.

Users were able to select from hundreds of variations of 58 core activities spanning the STAGE categories. However, some activities were used more frequently than others and were therefore more likely to be the activities used by those in our subsamples. One commonly selected activity, “Thx Thx Thx” (a thank task), asked the user to write down 3 good things that happened to them that day. One variation, “What Went Smoothly Today?,” instructed users as follows:

Think of three things about your day that went better than usual—maybe your commute to work was seamless, or you sent your kids off to school without a fight, or you simply had a little extra time for yourself. It can be anything—big or small. Jot them down and add a sentence or two describing why they made you feel grateful and what, if any[,] role you played in the experience.

Another activity, called “Savor the Small Stuff” (a savor task), instructed users to set aside a few moments to give their full attention to a sensory or cognitive experience. One variation, called “Smell the Roses,” instructed users thusly:

Indulge each of your senses by savoring something that’s right in front of you. For example, instead of walking past the local park in your neighborhood, sit down on a bench and be mindful as you take in the surroundings. What do you hear? Are there sights you’ve never noticed before? What does the air smell like? You can also practice mindfulness while savoring an indulgent dessert, or while looking at some of your favorite photos from good times past. How did you feel at the time? What did you talk about? Just be in the moment.

“I Think I Can” (an aspire activity) combined goal setting with behavioral activation research to get the user working on a goal. One variation of this activity, called “Mission: Possible,” instructed the user to

Think hard and narrow in on a goal that will be reasonable to achieve this week. No more excuses—aim to complete it this week, but make sure you pick something that terrifies you just a bit. Look at whatever’s cramming your ultimate to-do list, from a home renovation project to updating a blog or website or charging after new business. Then, decidedly look that fear in the eye, and without blinking, roll up your sleeves and get to the task at hand.

While a few activities on Happify could be done sitting at a computer (meditation, for example), many activities asked users to try a new behavior in their everyday lives, then report back on how it went. Users described what they did and how they felt about it in a textbox. Some activities specifically asked users to do the activity on Happify (eg, if a user was supposed to write down 3 good things that happened to them before bed, then Happify asked them to actually enter those three things into the site or app). Others, however, didn’t ask for the results of the activity itself (eg, if a user wrote a letter to someone expressing their gratitude, the letter itself would not go into the textbox, just their reflection on the experience of writing and delivering the letter). The text we had from users, therefore, was a mixture of words they used when doing activities and words they used when talking about their experiences with activities.

### Well-Being Assessment

Our primary outcome was well-being, which, consistent with current thinking on subjective well-being, we split into two components: positive emotion and satisfaction with life [28]. Due to the proprietary nature of Happify, a new measure (the Happify Scale) was developed and validated by the last author (AP) to measure well-being in users. Users were prompted to take the well-being questionnaire the day after registering, and again every 2 weeks after that.

The positive emotion subscale of the Happify Scale was developed based on the Positive and Negative Affect Schedule [29], which is a self-report survey that measures the extent to which a person has experienced a variety of both activated (high arousal) and deactivated (low arousal) positive and negative emotions. We shortened the survey for practical purposes to 4 sets of emotions: (1) joyous, exuberant, inspired, and awestruck, (2) serene, grateful, and relaxed, (3) sad, guilty, and lonely, and (4) angry, anxious, and afraid. For example, a user would be asked “In the past month, how often have you felt joyous, exuberant, inspired, or awestruck?” We added the 2 positively valenced items to the 2 negatively valenced items, reversed scored, to generate the positive emotion measure. In an internal validation study on a sample of 559 participants recruited via Amazon’s Mechanical Turk (Amazon.com, Inc, Seattle, WA, USA), the positive emotion subscale had acceptable internal consistency (alpha = .72) and was strongly positively correlated with the Positive and Negative Affect Schedule (r=.76, *P*<.001).

We modeled the life satisfaction subscale of the Happify Scale after the Satisfaction With Life Scale [30] but adjusted it to ask users about satisfaction with different domains of their life, including work, leisure, and relationships. For example, a user would be asked “How satisfied do you feel with the relationships in your life?” We computed this score as a simple sum. In the previously mentioned internal validation study, the life satisfaction subscale had acceptable internal consistency (alpha=.88) and was strongly correlated with the Satisfaction With Life Scale (*r*=.80, *P*<.001).

### Study 1

Previous research has found that continued practice of happiness activities leads to better outcomes among happiness seekers compared with those who do not practice on a regular basis [16]. Therefore, we hypothesized that usage is tied to improvement, such that users experience higher well-being scores during periods when they use the site more and lower well-being scores during periods when they use the site less. We chose to focus our analysis on the positive emotion subscale of the Happify Scale, as previous research has shown that life satisfaction is relatively stable in the short term, while positive emotion is more susceptible to change [30,31]. Consistent with previous research, we also expected to see greater improvement in users with lower well-being to start.

#### Study 1 Methods

##### Participants

The sample comprised 152,747 users who completed at least two well-being assessments. Users were asked to complete a well-being assessment the day after registration, but some users did not return after their first visit and therefore were never offered the assessment. Others chose not to complete the assessment but kept using the site. Therefore, the sample contained users who were moderately interested in the platform and who were interested in tracking their own well-being.

To examine the possibility of sample bias as a result of excluding participants with <2 assessments, we compared those who completed ≥2 assessments (n=152,747) with those who completed only 1 (n=568,205) on the measure of positive emotion. Table 1 shows the results. There was no statistically significant difference between users who completed 1 assessment and users who completed ≥2, and the effect size for the difference between the means was very small per Cohen *d* guidelines [32]. However, when it came to positive emotion, we did not have the ability to compare users who completed any number of assessments with users who completed none. It is still possible (perhaps even likely) that our sample was not representative of the overall user base in terms of well-being levels.

We also compared these 2 groups on demographic variables using chi-square tests and, for the most part, found statistically significant but practically small differences (see Table 2). Specifically, compared with users who did not complete ≥2 assessments, our sample had more women, fewer people aged 18–24 years and more people aged 35–44 years and 45–54 years, fewer students and more people who were employed, more people without children, and fewer people with children ≥19 years old and with children of different ages. However, most of these differences are in the 1% to 3% range, and were likely significant only because of the very large sample size. The only substantial, and possibly quite important, difference was in the age of the ≥2 assessment sample. Users who completed ≥2 assessments were significantly older than the overall user base, with 6% fewer people in the 18- to 24-year age range.

**Table 1:** Baseline differences on positive emotion measure between the study 1 Happify user sample (completed ≥2 assessments) and users who did not complete ≥2 assessments.

Number of assessments	No.	Mean score^a^	SD	*t*	*df*	*P* value	*d*
1	568,205	38.75	19.80	3.39	720950	.99	.00
≥2	152,747	38.56	19.38				

^a^Scored on a scale of 1–100 in the Happify Scale.

**Table 2:** Differences in demographic variables between the study sample (≥2 assessments, n=1,925,376) and those not included in the analysis for study 1 (1 assessment only, n=152,747).

Characteristics		1 Assessment, % (n)	≥2 Assessments, % (n)	χ^2^	Cramer *V*	*df*	*P* value
**Sex**				1371.56	.05	2	<.001
	Male	13% (19,857.11)	10% (192,537.60)				
	Female	87% (132,889.89)	90% (1,732,838.40)				
**Age range (years)**				4075.98	.07	5	<.001
	18–24	20% (30,549.40)	13.9% (267,627.26)				
	25–34	30% (45,824.10)	30% (577,612.80)				
	35–44	24% (36,659.28)	28% (539,105.28)				
	45–54	17% (25,966.99)	19% (365,821.44)				
	55–64	8% (12,219.76)	8% (154,030.08)				
	≥65	1.5% (2291.21)	1.5% (28,880.64)				
**Employment status**				1804.80	.12	5	<.001
	Retired	3% (4,582.41)	3% (57,761.28)				
	Self-employed	12% (18,329.64)	12% (23,1045.12)				
	Unemployed	6% (9,164.82)	6% (115,522.56)				
	Student	14% (21,384.58)	11% (21,1791.36)				
	Employed	57% (87,065.79)	62% (1,193,733.12)				
	Homemaker	7% (10,692.29)	7% (134,776.32)				
**Parental status**				1714.74	.05	5	<.001
	Children ≥19 years	7% (10,692.29)	5.4% (103,970.30)				
	Children 13–18 years	2% (3,054.94)	2% (38,507.52)				
	Children 0–12 years	5% (7,637.35)	5% (96,268.80)				
	Children of different ages	5% (7,637.35)	4% (77,015.04)				
	No children	15% (22,912.05)	18% (346,567.68)				

In summary, when considering to whom this research is generalizable, it is important to remember that the subsample we drew from was biased in one key way that may limit generalizability: our participants were older than the overall Happify user base. Furthermore, our sample may be biased when it comes to users’ well-being levels; the data available suggested not, but we did not have data for users completing no assessments. Based on previous research, it is likely that those users were different from our sample in some way.

##### Baseline Well-Being as a Moderator

On a scale of 0–100, the average positive emotion score among study 1 users at baseline was 39.03 (SD 19.45) and average life satisfaction was 52.00 (SD 22.78). However, previous research suggests that there are two distinct types of happiness seekers: those who are relatively distressed and those who are relatively nondistressed [19]. Other work replicates this 2-cluster structure, typically derived from a positive emotion measure, a life satisfaction measure, and a depression measure, and suggests that these different groups may respond differently to happiness interventions [17]. Specifically, some evidence suggests that happiness seekers who are more distressed may experience greater benefit [18]. Therefore, following a similar procedure used in previous work clustering happiness seekers, we performed a 2-step cluster analysis in IBM SPSS (version 19, IBM Corporation) using baseline positive emotion and life satisfaction scores to sort participants in our sample. Previous research has found this approach to be robust for use in large datasets [33,34]. Although we did not have a measure of depressive symptoms, we hypothesized that using 2 of the 3 measures used previously would still yield a 2-cluster pattern of division, with one group showing overall higher than average well-being and the other showing overall lower than average well-being.

Even without a measure of depressive symptoms, we found the expected cluster structure. As anticipated, the model yielded two distinct types of users in the sample: low well-being (n=69,474), whose mean scores for positive emotion (23.64) and life satisfaction (32.63) were lower than the sample average; and high well-being (n=83,273), whose mean scores for positive emotion (51.05) and life satisfaction (68.01) were higher than the sample average. Multimedia Appendix 1 displays the silhouette diagram for the model [35], which describes the model fit as good. We used this clustered variable as a moderator in our effectiveness analysis to see whether Happify affected distressed users differently from nondistressed users.

##### Analytic Strategy

We analyzed data in IBM SPSS using a multilevel modeling procedure originally designed for use in diary data, where multiple assessments on both the independent and dependent variables are taken for each individual participant [13]. Multilevel modeling is an advanced form of linear regression that is ideal for assessing longitudinal data because it is able to use however few or many assessment points a user has provided (in other words, it does not discard users with missing data and instead plots a line for them using whatever data they gave).

In this particular variation of multilevel modeling [36], there is—like any multilevel model for an intervention—a main effect for time, which shows how well-being changed over the course of the 8-week intervention period. We included time in the model because many intervention studies explore psychological changes in this way: incrementally, in sequence, over time. Furthermore, including time is important because it controls for the possibility that the relationship between usage and outcome was due only to the passage of time [36]. However, since a main effect of time was uncontrolled, and is subject to criticisms such as regression to the mean and confoundedness with usage and dropout rate, we were especially interested in analyses that look at well-being as a function of usage. When analyzing usage, we separated within-person and between-persons variation, and we assessed both between- and within-person terms separately in the model. The model, then, yielded estimates for time, within-person variation (ie, whether a person’s well-being varied was a function of their using Happify more or less), and between-persons variation (ie, whether users with an overall pattern of high usage differed from users with an overall pattern of low usage) with well-being as the dependent variable. Therefore, analyses involving usage (within or between) focused on the short-term impact of usage—during each individual 2-week window—rather than taking place over the entire course of the 8-week intervention period.

For between-persons terms, we compared people who tended to use the app more with people who tended to use the app less. Usage is continuous, not categorical, in this term. A significant between-persons term would indicate that an overall pattern of higher usage predicts higher well-being. Between-persons differences in overall usage patterns were not of primary interest to us but, rather, were important potential confounding variables to control for as we explored the impact of within-person variation. For the within-person terms, a dose-response line could be calculated for each individual participant, not over time, but over levels of usage. It examined the relationship between usage and mood for that individual. Analysis of within-group effects provided a way to see how a person did at different “dosages,” and the focus was on their change within-person, at these different dosages, rather than on differences between people who got one dose or another. Such an analysis is not as vulnerable as a between-persons comparison based on usage (splitting by heavy users vs light users) would be, as there would likely be systematic differences between the people in the 2 groups. Because a participant was being compared with him- or herself, concerns about differences between users became less salient.

In smaller subsamples, the participants’ individual lines could be visualized in a “spaghetti plot,” which shows individual differences in the role of usage on well-being. Spaghetti plots are a useful adjunct to a statistical model, as they help to visualize the extent to which the model’s overall slope is representative of the slope of each individual in the sample. More simply, they indicate whether the overall slope is representative of what is happening in the sample. The analysis set well-being (positive emotion and life satisfaction were examined separately) as the dependent variable and usage as the predictor.

All participants had at least two well-being assessments, but some had up to 5 assessments, spanning 8 weeks. The 8-week assessment period began from the time users completed their first well-being assessment. For any time that well-being was assessed, we calculated a “usage” variable, which we operationalized as the number of visits to the site in which the user completed an activity that took place between the last assessment and the current one. The average number of activities completed prior to the first assessment was 5 (SD 5.11). Additionally, we used a baseline clustering for well-being as a moderator (see above). We tested the predictive power of usage, as well as the potential interaction of baseline well-being with usage, with between- and within-persons variation separated. The analysis, therefore, yielded traditional estimates of how much the average person improved (a between-persons approach), but also looked at how usage variation for each individual predicted his or her well-being (a within-person approach), generatin a dose-response relationship. In short, it asked “For any given person, how was their well-being during time periods that they used Happify more, and during time periods where they used Happify less?”

#### Study 1 Results

Table 3 contains the descriptive statistics for the positive emotion measure, as well as for usage, across each of the time points. Positive emotion increased by 10.47 points (10.47%) over the course of the 8-week study period. Usage, which started relatively high (about 5 visits per week), decreased over time and by 8 weeks was between 0 and 1. The sample size for positive emotion changed over time as users dropped out, but because usage was observed rather than self-reported, data on usage were available for everyone in the sample, regardless of compliance with the well-being assessment.

**Table 3:** Positive emotion^a^ and usage among a sample of Happify users over the course of 8 weeks.

Time point		No.	Mean	SD
**Positive emotion scores**				
	Baseline	152,747	38.56	19.38
	2 weeks	148,740	42.46	19.68
	4 weeks	52,177	45.29	19.80
	6 weeks	25,435	47.46	19.73
	8 weeks	15,140	49.03	19.63
**Usage (visits/week)**				
	Baseline	152,747	5.19	5.11
	2 weeks	152,747	4.39	11.40
	4 weeks	152,747	2.06	8.26
	6 weeks	152,747	1.25	6.45
	8 weeks	152,747	0.85	5.15

^a^Scored on a scale of 1–100 in the Happify Scale.

There was a main effect for time that echoed the observed mean increases in positive emotion. As time passed, positive emotion improved at a rate of about 1.38 points per week for the average user (estimate 1.38, SE 0.01, *t*_122,455_=113.60, *P*<.001, 95% CI 1.36–1.41). This suggests an average overall improvement of 11.04 points, or about 27%, over the course of 8 weeks.

There was also a significant impact of usage on overall well-being compared with users who did not use the platform as often. On average, high-usage users experienced more positive emotion (estimate 0.20, SE 0.02, *t*_85,929_=11.63, *P*<.001, 95% CI 0.17–0.23). The significance of this term suggests the importance of including it as a control variable. However, due to the way usage was measured (segmented into 2-week chunks, rather than being cumulative), the estimate yielded by this term is not meaningful or interpretable for practical purposes.

The results for the within-person terms revealed that for any given user, more usage predicted more positive emotion and less usage predicted less positive emotion (estimate 0.09, SE 0.01, *t*_6047_=9.15, *P*=.001, 95% CI 0.07–0.12). This estimate predicted that a given user would report positive emotion that would be 1.26 points higher after a 2-week period when they used Happify daily than after a week when they didn’t use it at all. On a week-to-week basis, it seems that users got more out of the site during weeks when they used it often, and less when they used it rarely.

**Figure 1:**
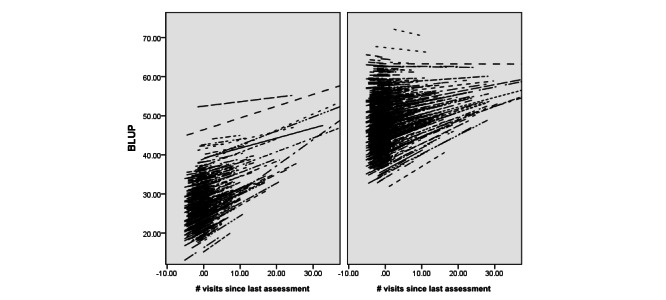
Spaghetti plot illustrating the impact of usage (number of visits since last assessment, grand mean centered) on positive emotion for Happify users with low baseline well-being (left) and high baseline well-being (right). Positive emotion is represented with best linear unbiased prediction (BLUP) scores. Illustrated using a randomly selected subsample of n=1505.

There was also a significant between-persons usage × baseline well-being interaction such that people with lower baseline well-being and who used the site more often experienced the most improvement compared with high well-being users, or low well-being users who did not use the site often (estimate 0.20, SE 0.02, *t*_85,929_=18.60, *P*<.001, 95% CI 0.18–0.22). Both baseline well-being level and usage seemed to interact to determine improvement. Although, for reasons described above, the estimate yielded here is not meaningful or interpretable, the results can be seen in Figure 1, which shows spaghetti plots for low well-being users (left) and high well-being users (right). Due to computer memory limitations in rendering the graph, we created the plot on a randomly selected subset of the data (n=1505). There were no statistically significant differences from the overall sample on baseline well-being or demographic variables, and the pattern of results observed in the sample was the same. The x-axis is usage (number of visits since the last assessment), grand mean centered. The y-axis is a best linear unbiased prediction [37], which is intended to capture random effects for positive emotion. Each line on the plot represents the dose-response curve for an individual user; longer lines indicate users with greater variability in usage. The overall positive relationship between usage and well-being is visible in both plots. Furthermore, the graphs indicate that, although more usage seems to lead to higher well-being for users starting with low well-being, the benefits of usage on well-being seem to level off at higher usage levels for some users in the group with higher starting well-being.

There was not a significant within-person usage × baseline well-being interaction. Low well-being or high well-being participants did not show differing levels of sensitivity to usage (*P*=.28). The relationship between usage and well-being was the same regardless of a person’s baseline well-being.

#### Study 1 Discussion

The results suggest overall improvement across users of Happify regardless of baseline well-being, with higher usage generally associated with higher well-being across the board. The average person using the site improved, with more improvement the more they used it. However, that improvement seems to be greater among those who started with lower well-being, especially on the measure of positive emotion. Thus, users with more room to grow due to their low well-being experienced greater changes in well-being when using the site than did their high well-being counterparts.

It is important to consider some limitations to the study design, and to temper interpretation of the findings accordingly. The first is the bias we observed in our sample. Users who completed ≥2 questionnaires, a requirement for inclusion, were substantially older and may have otherwise been different in ways we could not assess from users who completed only 1 questionnaire. While we did not know the well-being level of the great majority of users who were excluded from the sample (they did not complete even 1 well-being measure), other studies have suggested that dropouts may be biased toward lower well-being, and we also observed bias toward an older demographic. We also observed substantial drop off over the course of the study, with only 10% of users completing all assessments; while these dropout rates are typical in naturalistic Internet intervention, they also press the limits of what regression can reliably do to accommodate missing data. We also acknowledge that our operationalization of usage—the number of visits to the site in which an activity was completed—is only one of many ways to quantify usage. For the purposes of our analysis, it was important to quantify usage every 2 weeks so that each 2-week period could be analyzed separately. However, in an analysis that looked at change over time continuously, usage could be conceptualized cumulatively. For example, a user could have an ongoing tally of “number of activities completed” that grows over time. This type of approach would be beneficial for looking at total usage, something we were unable to do in this study.

Nevertheless, we were able to observe these effects outside of a controlled research setting, which was one of our goals, since control groups are often impractical in large, naturalistic data sets. The use of multilevel models offered us a way to sidestep some (but not all) problems with an uncontrolled design. Within-person terms of the model (focusing on whether usage at different time points led to changes in well-being for any given person) are less vulnerable to traditional criticisms of an uncontrolled study. However, between-persons terms (finding that some users do better than others) are still vulnerable to concerns about sample bias. This approach to evaluating effectiveness, while not at all the same as conducting an RCT, may allow researchers to get an estimate of effectiveness when an RCT is not possible.

### Study 2

Study 1 provided evidence that users who interacted with the site more often also reported higher levels of well-being. However, it did not suggest the *ways* of engaging with the site that were associated with improvement. An important means of addressing this question is by analyzing the language expressed by users as they complete activities. By using big data techniques to determine the topics associated with user improvement, we can provide insight to users’ spontaneous behavior; that is, what behaviors are beneficial when users are free to engage with an intervention however they wish. Because our methods were data driven, they allowed the data to speak for themselves. We had no specific, pre hoc hypotheses about our results. Our goals were to develop insight into the kinds of spontaneous engagement that are associated with successful outcomes in large, open Internet well-being interventions such as Happify. Looking only at users who were engaged to begin with, we isolated the language associated with improvement, giving insight into the maximally effective ways to be engaged.

#### Study 2 Methods

##### Materials

For all linguistic analyses, our dependent variables of interest were the same pair of self-report surveys used in study 1. We derived predictor variables from task-based text that participants wrote over the course of their time (see the general Methods section above for more description of the activities on the site) using natural language processing methods described below.

##### Participants

To construct stable outcome variables based on the well-being scales, we limited linguistic analysis to users who completed the scales at least twice and who had a span of at least 30 days between first and last self-report. Table 4 lists differences between those who met these criteria and those who did not. Note that demographic information was not available for all participants with ≥500 words, so the users analyzed here are only a subset of those used in our analysis. In addition, because large amounts of an individual’s language are required for reliable language analysis [38], we only considered users who wrote at least 500 words across all free-text tasks. This left us with a final sample of 10,818 users. Participants in the sample used Happify over an average of 168 days, and they wrote an average of 51.23 words for each task they completed.

**Table 4:** Differences in demographic variables between the sample (<500 words, n=2,073,333) and those not included in the analysis for study 2 (≥500 words, n=4790).

Characteristics		<500 words, % (n)	≥500 words % (n)	χ^2^	Cramer *V*	*df*	*P* value
**Sex**				169.19	.02	2	<.001
	Male	12% (248799.96)	9% (431.10)				
	Female	87% (1803799.71)	90% (4311.00)				
**Age range (years)**				381.55	.04	6	<.001
	18–24	19% (393933.27)	19% (910.10)				
	25–34	30% (621,999.90)	37% (1,772.30)				
	35–44	24% (497,599.92)	24% (1,149.60)				
	45–54	17% (352,466.61)	14% (670.60)				
	55–64	8% (165,866.64)	6% (287.40)				
	≥65	2% (41,466.66)	1% (47.90)				
**Employment status**				155.44	.03	5	<.001
	Retired	3% (62,199.99)	2% (95.80)				
	Self-employed	12% (248,799.96)	12% (574.80)				
	Unemployed	6% (124,399.98)	8% (383.20)				
	Student	14% (290,266.62)	16% (766.40)				
	Employed	58% (1,202,533.14)	52% (2,490.80)				
	Homemaker	7% (145,133.31)	7% (335.30)				
**Parental status**				8951.68	.03	5	<.001
	Children ≥19 years	7% (145,133.31)	7% (335.30)				
	Children 13–18 years	2% (41,466.66)	3% (143.70)				
	Children 0–12 years	5% (103,666.65)	10% (479.00)				
	Children of different ages	5% (103,666.65)	5% (239.50)				
	No children	15% (310,999.95)	45% (2,155.50)				

Table 5 shows descriptive statistics for the sample, as well as an analysis of baseline differences between users in our sample and the remaining users in the user base. Users in the study 2 sample had significantly higher well-being at their first assessment than did users who did not write ≥500 words. Not surprisingly, this subsample is not a random subset of the overall user base—a very specific set of users engaged with the site frequently enough to yield the amount of text needed by this analysis.

**Table 5:** Baseline characteristics for the study 2 sample on both dependent variables, and analysis of the difference between users in the study sample (who wrote ≥500 words) and users who wrote <500 words.

Dependent variables		No.	Mean score^a^	SD	*t*	*df*	*P* value	*d*
**Positive emotion**								
	<500 words	710,348	39.43	19.97	–47.41	721,164	<.001	.46
	≥500 words	10,818	48.63	20.11				
**Life satisfaction**								
	<500 words	710,348	52.31	23.36	–44.39	721,164	<.001	.42
	≥500 words	10,818	62.39	24.26				

^a^Scored on a scale of 1–100 in the Happify Scale.

This sample was clearly not randomly taken from the population of Happify users, and is therefore likely not representative of the whole user base. However, focusing specifically on highly active users let us explore the *kind* of activity associated with improvement. Even among highly engaged users, there was variance in improvement experienced. Therefore, simply using an intervention often may not be enough for it to be effective. There are likely specific behaviors and psychological orientations associated with improvement. Our linguistic analysis used data-driven techniques to reveal these factors.

#### Procedures

##### Text Preprocessing

Some Happify tasks contained multiple text fields. We combined all text for a given task instance into a single document for topic modeling. Hard returns were replaced with a “<newline>” placeholder. Tokenization, feature extraction (other than topic modeling), regression, and correlation analysis were performed in Python version 2.7 (Python Software Foundation).

##### Topic Modeling

We clustered users’ free text using a latent Dirichlet allocation (LDA), a topic modeling technique [39]. The LDA technique assumes that documents (in our case, text from a single task instance) comprises a combination of topics, and that each topic is a cluster of words. Using the words found in each document, the makeup of each topic is estimated using Gibbs sampling [40,41]. We used the Mallet implementation of the LDA algorithm [40] to produce 200 topics, adjusting the alpha parameter (alpha=5) to tune for fewer topics per document due to each document’s short length compared with typical applications of LDA (eg, encyclopedia or news articles). Previous work by members of our research group [42] used a larger number of topics, 2000, but the task-directed language in the study 2 data set led to reduced variability in language, able to be captured with a smaller number of topics. To use topics as features, we calculated the probability of a user’s topic usage, p(topic | user), using LDA outputs and user word probabilities (see [41] for details). This gives a 200-dimensional vector representing the language of each user, where each dimension maps onto a discrete topic word cluster.

##### Factor Analysis of Topics

LDA produces topics that can be strongly intercorrelated over users [43,44]; that is, if topics are correlated, when users write about certain topics, they tend to also write about other specific topics that are related to them. For example, a user who writes about cooking is more likely to also write about dessert than about schoolwork, even though “cooking” and “dessert” constitute 2 separate topics.

Multimedia Appendix 2 shows the intertopic correlation matrix and scree plot for the 200 topics. While most topic correlations were weak or very weak, 41 topic pairs exhibited moderate correlations, and 6 topic pairs exhibited strong or very strong correlations. To account for topic correlation, we ran exploratory factor analysis on topic scores using varimax rotation. Based on the scree plot, we decided to use a 50-factor solution that exhibited a close fit (root mean square error of approximation of 0.01807) explaining 32.5% of overall variation. The resulting factors may be viewed as patterns of user behavior.

In summary, the LDA topics clustered together aspects of messages that likely had some similarity. The factor analysis clustered together topics that tended to co-occur within the same user. This difference allowed us to answer similar but distinct questions: (1) What do people write about that is related to effective engagement with the intervention? (2) What types of general user behavior are related to effective engagement with the intervention?

##### Outcome Variables

We constructed ordinary least squares regressions with time (days since first response) as the independent variable and an overall well-being measure constructed by aggregating the positive emotion score and the overall life satisfaction score as a dependent variable. Our outcome for each participant was the ordinary least squares regression slopes of users’ total score (positive emotion plus life satisfaction) change over time. Positive emotion and life satisfaction subscales were correlated at *r*=.61, and analyzing them separately did not produce meaningfully different results (see Multimedia Appendix 3). Therefore, for simplicity, we combined these variables into a single score of subjective well-being [45].

##### Correlation Analysis

Using the technique of differential language analysis [9], we correlated users’ well-being change over time with language rates on two levels: LDA topics and user-level factors.

Because this analysis involves multiple, independent tests, which increases the possibility of type I error, we applied Benjamini-Hochberg false discovery rate correction to our results, which adjusts *P* values based on the number of tests run [46].

A positive correlation indicated that the language feature was associated with improvement in well-being. If a particular topic positively correlated with the outcome, then it meant that writing more often about that topic was associated with higher levels of improvement in well-being over time. Because of our specific interest in users’ improvement, we only considered positive correlations between language features and outcomes.

#### Study 2 Results

##### Topics

**Figure 2:**
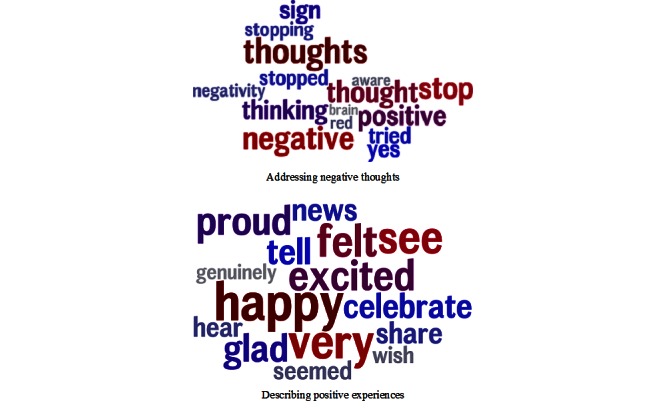
Example topics predicting increased well-being.

A total of 14 topics significantly predicted increased well-being. The topics with the strongest relationship tended to be about directly engaging with negative thoughts and emotions, but some also included descriptions of positive experiences. Figure 2 displays examples of each kind. All topics with a significant effect are included in Multimedia Appendix 4.

We reran the topic analysis only for users with initially high well-being to address the possibility that the results were influenced by a ceiling effect. The results are similar to those of the overall sample (see Multimedia Appendix 5).

##### Factors

**Figure 3:**
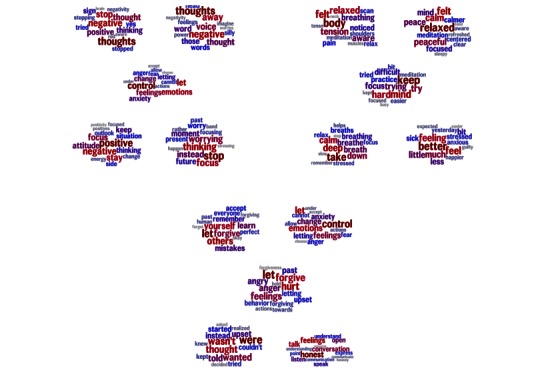
The 5 highest-loading topics within the 3 factors predicting improved well-being. The top left factor contains topics about negative thoughts (restructuring negative thoughts). The top right factor contains topics about dealing with anxiety (controlling anxiety). The bottom factor contains topics about past experiences, and conflicts and interactions with other people (coming to terms with interpersonal strife).

A total of 3 factors significantly predicted increased well-being, and they also primarily centered on engaging actively with negative experiences. For ease of interpretability, we labelled these factors with 3 distinct aspects of this process: (1) restructuring negative thoughts, (2) controlling anxiety, and (3) coming to terms with interpersonal strife. These labels, while open to interpretation and derived post hoc (not from the language analysis), support the general pattern of an actively positive orientation toward life difficulty. Figure 3 illustrates the 5 highest-loading topics for these 3 factors. These factors describe general user behavior, providing a more contextualized view of the language associated with successful engagement.

#### Study 2 Discussion

Automatic language analysis revealed the topics and factors associated with successful engagement with Internet well-being interventions. Of note is that increased positive affect and life satisfaction were associated with negatively valenced topics containing such words as “negative,” “anxiety,” and “worrying.” Among those who were already highly engaged, the most successful users addressed their unpleasant thoughts, anxieties, and difficulties. This result is consistent with previous, experimentally based theories. Cognitive therapy techniques focus on intervening in maladaptive thought patterns [47]. Also, previous research has shown health and well-being benefits specifically from writing about negative experiences [48,49].

One reason why users may have found themselves discussing negative content was the structure of Happify. As discussed above, users’ experiences on Happify are framed by tracks, or sets of activities that are built around a common goal or theme. The overwhelming majority of the user base chose problem-focused tracks such as “conquering negative thoughts” and “cope better with stress.” Therefore, while the tasks they did were quite varied and were generally focused on positive emotion, they also referred to negative aspects of the user’s life. In that sense, we may also have been detecting adherence and compliance wherein users who stayed on point with the goals of the track by discussing negative content benefitted the most.

Successful users also focused on a variety of positive emotional experiences. Notably, many of these experiences referred specifically to positive emotions, such as pride, enjoyment, appreciation, and celebration, which are reactions to specific objects or events rather than general moods.

In general, users who increased their well-being wrote frankly about their unpleasant or unadaptive thoughts and feelings, addressing these negative experiences with direct strategy. However, they did not write solely about unpleasant things, and therefore they balanced both positive and negative topics. These qualitatively induced conclusions are based on real-world data and capture important trends in people’s spontaneous attempts to increase their own well-being. In other words, even among users who engaged strongly with the interventions, there was variance in how much benefit they received. Users who put in the effort of working through their unpleasant emotions and thoughts had the greatest improvement in well-being. Probably due to the relative sparseness of language for each user, correlations between these topics and outcomes were rather weak (see Multimedia Appendixes for details). This fact reveals another important feature of big data methods: they allow researchers to uncover subtle effects such as these that would have been hidden in smaller samples. Though small, these results help explain well-being improvement across a large number of people in an uncontrolled setting, and therefore potentially have dramatic real-world impact. Furthermore, the open nature of these platforms means that data will continue to accrue, allowing increasingly powerful and nuanced language analyses.

## Discussion

The goal of this study was to showcase the advantages of analyzing intervention data in a massive but uncontrolled dataset. Our goal was to try two novel methods for making sense of the chaos in uncontrolled and potentially overwhelming intervention data. In study 1, consistent with hypotheses and with previous research [16,18], we found that the average Happify user improved in well-being by about 11% over the course of 8 weeks. However, without a control group, it is difficult to infer that the intervention, and not the passage of time or other factors, led to the observed improvement. Thus, we also explored within-person dose-response relationships and found that users reported higher well-being during periods of time when they used Happify more frequently. While this type of design is weaker than its more controlled counterparts when inferring overall effectiveness, it does allow us to explore what happens to users while they use an intervention at a finer level of granularity, week by week, as their usage levels vary. It may also help us to determine what level of usage is optimal or whether, at some point, more usage stops being better.

The within-person analysis with usage predicting well-being week to week is interesting in that it looked not only at differences between users, but also differences within users at different dosages of the intervention. While this type of approach is typical in diary and experience sampling research, it is seen less frequently in intervention research. Baseline well-being was a moderator, such that users with lower starting well-being improved more. This is also consistent with previous work [17,18]. Using natural language analysis, a procedure only possible in large datasets, study 2 yielded a snapshot of what processes may be going on when users successfully practice activities on Happify. Rather than focusing solely on the positive, users appeared to be using happiness-focused activities as a way of working through problems. In some sense, this is surprising, because the activities in the STAGE model are all focused on positive experiences and cognitions. However, there is evidence that writing about the negative can help individuals create a meaningful narrative, which may also be happening [48].

When evaluating effectiveness, many researchers may avoid these kinds of data because the lack of a control group makes it difficult to establish the effectiveness of an intervention. To address this problem, we also examined within-person variation, watching how each user’s well-being varied as a function of their usage frequency. Control groups typically exist to account for systematic differences between participants in each group, as well as natural change over time. A within-person analysis is less subject to these concerns, since each data point comes from the same person. Using this approach, our results indicated that, for most users, usage and well-being went hand in hand, with greater usage during a given 2-week period predicting higher well-being at the end of that period. We were also able to include the effects of individual differences by visualizing these data using spaghetti plots. The plots indicated that, although a small number of outliers existed, the positive relationship between usage and well-being held true for most of the sample.

Another hesitation that many researchers might have is the inability to explore theoretical mechanisms. However, we were able to create insight into mechanisms with the use of linguistic analysis to identify topics that were more likely to be used by users whose well-being improved. Discussion of negative topics, especially negative thoughts, was associated with better outcomes. Users acknowledging and engaging with their anxieties and unadaptive cognitive patterns reaped benefits. While these results are ultimately just correlational, they provide a descriptive snapshot of what distinguishes effective use of the intervention from less effective use. Furthermore, as research converges on the types of language use that are most strongly related to improvement, this kind of fine-grained data can provide intervention designers with feedback on how to present an activity. For example, if focusing on negative topics continues to emerge as a common factor among people who benefit from Happify, then Happify would do well to revise activity instructions to encourage users to focus on negative topics.

The nature of the dataset also carried specific advantages over more structured intervention research study designs. Most apparent is the large sample size, which was several orders of magnitude greater than all but the most ambitious randomized studies. This large sample size allowed us to generate parameters with a high amount of confidence, including the standard errors generally in the 0.01–0.03 range, with tight 95% confidence intervals. It also allowed us to do a moderator analysis that examined differences between people whose baseline well-being was high or low, without hindering our ability to detect effects. Often, moderator analyses are reserved for meta-analyses, where the data of multiple studies are available. Also, automatic language analysis requires large amounts of data to provide valid results, rendering it an impossible tool in most controlled intervention studies. Furthermore, the very nature of these datasets means that they are continually expanding. As the amount of data increases, so too does the ability of researchers to measure and isolate nuanced effects.

The dearth of experimental control over variables within our data, while in some ways a liability, also provided access to important research questions. In the real world, users are free to engage with interventions whenever and however they wish; these variables are therefore important to consider in studying general effectiveness. Our data describe participants’ spontaneous behavior; that is, their levels of engagement in the midst of their everyday lives and their freely chosen strategies when using the intervention. We therefore provide results that are not only externally valid but also engage with variables that would be “controlled away” in many other designs.

Many authors have noted the need for new methodologies to accommodate the rapidly changing landscape of Internet intervention research [2,3]. We modeled the use of some alternative strategies that yield valid information about outcome and process in Internet interventions. Thus far, we have focused on the benefits of using real-world intervention data. It is, however, worthwhile to discuss some limitations as well.

### Limitations

Real-world interventions are constantly changing by releasing new content, revamping existing features, and providing interactions with other users via discussion forums, and that presents a challenge to researchers who are trained to keep as many factors as constant as possible [50]. Furthermore, the effectiveness evaluation approach we used relies on within-person variation in usage, which is not as clean an independent variable as randomly assigned group membership. Users are self-selected on several levels from their decision to try Happify at all, and even more so in their continued use. The nature of our analytic strategy—using usage, text input, and well-being data—restricted us to users who provided that type of data in sufficient quantity. For study 1, we were limited to users who completed well-being assessments. For study 2, we were limited to users who wrote ≥500 words. For both studies, we had demographic data only for users who reported them. In all of these cases, many, many users were eliminated from our sample due to insufficient data. We can’t analyze data that we don’t have and, as a result, our samples were biased. We took some measures to explore the nature of that bias, but missing data makes it difficult to be sure that we understand every potential way that the data are not representative of the overall user base.

While temporal precedence of usage behavior compared with well-being scores allowed for some measure of ability to infer causality, there were also potential third variables that could have influenced both usage and well-being. One major concern was the role of dropouts in our findings. Dropouts are common, and dropout rates are high, even in controlled research studies where some incentive for participation may be provided [51]. In a consumer environment, where users are customers and it is a buyer’s market, the frequency of dropout becomes even greater [51]. This was apparent in our sample, where the rate of attrition mirrors that observed in other published analyses of uncontrolled intervention datasets. While high attrition rates are certainly cause for concern and caution when interpreting findings, they do not render a study without value [51]. Nevertheless, it is important to consider potential sources of bias that may be introduced with so large a percentage of the sample missing and, as much as possible, to account for those missing data using appropriate statistical approaches.

We made some attempts to uncover any possible differences between people who did and those who did not engage in Happify enough to meet our inclusion criteria, and we did find some key differences between users who were highly engaged (completing ≥2 assessments or writing ≥500 words) and those who were not highly engaged. Available demographic data were, however, limited and many other factors that we did not measure may have played a role in dropouts. Therefore, there remain other potential biases in our samples that are difficult to determine. The linguistic analysis has its own limitations in terms of control. Due to the large amounts of language required for analysis, we were unable to observe the specific pattern of changes in language use over time as a result of intervention use.

We also acknowledge that our mode of measuring well-being using two self-report measures, based on the subjective well-being model, is just one of many possible ways to measure well-being. Self-report measures exist for other conceptualizations, including psychological well-being [52] and mental well-being [53]. Beyond that, it is possible to measure emotions on a more day-to-day basis using experience sampling methods, a methodology that is becoming more and more possible as more people begin to carry mobile phones. Furthermore, recent technological advances in wrist-worn technology bring variables such as heart rate variability within the grasp of researchers. There are many possible frontiers for advancing the assessment of well-being in big data research; ours was just a first step.

Another issue with real-world intervention data is a preponderance of statistical power. Study 1 analyzed the data of over 150,000 participants; at that sample size, nearly everything is significant, and statistical significance therefore cannot be relied on as an indication of which findings are “important.” The potential for overpowering is just as great in the linguistic analysis, where many effects, while statistically significant, were very small. As linguistic analysis of this kind is relatively new, it is hard to know what constitutes a meaningful effect. Future research is needed to explore the predictive power of word and topic usage on future behavior to clarify how much this particular process matters for well-being.

Finally, data from real-world interventions are problematic in that they have a reputation for being inaccessible to researchers. It is not always of interest to a company to produce scientific research, and it is not always of interest to researchers to have conversations with companies. We would encourage both groups to reach out to one another and forge mutually beneficial relationships. Published research using company data can strengthen the company’s legitimacy. Researchers can access massive, free (to them!) datasets containing outcomes that often are not available in more controlled settings, which is, we hope we have convinced the reader, worth pursuing.

### Ethics: An Outstanding Issue

Although we aimed in this study to help establish some new and interesting approaches to analyzing big intervention data, there are some issues that our research raises, but does not address. One important topic for further discussion is the ethics of consent in the context of commercial products. Is passive consent by endorsing a user agreement enough, or should there be more active consent procedures? For example, one might imagine implementing a “use my data for research” dialogue box on each user’s profile that is opt-in, so data are only analyzed for users who have considered the risks and decided to make their data available. At the same time, doing so would greatly restrict both the sample size and potentially the sample’s validity; what systematic differences might exist between users who do not opt into research? Wouldn’t a user need to be relatively engaged in the first place in order to opt in to research? And, if so, wouldn’t it be impossible to examine differences between users who engage highly and users who do not, since researchers would not have access to the data of those who do not? It seems clear that in a research context, where users would be randomly assigned between conditions and one or more of those conditions may be intended as “inert,” informed consent is a necessity. However, more discussion is needed to develop standards for consent in a context that lacks random assignment, where everyone gets the “best” possible product, and where the purpose of the site is not to collect data or perform an experiment, but to deliver that product to the public.

### Conclusions

We have provided some novel insight into the relationship between general usage of a Web- and app-based platform, as well as the use of specific topics, and change in well-being. We have not, of course, provided an exhaustive or comprehensive review of big data methodologies that might be appropriate for Internet interventions. However, we hope we have effectively argued that analytic methods for unstructured intervention data are needed; without them, entire, massive, naturalistic datasets will remain out of reach. We also hope that our initial attempt at exploring such an unstructured dataset will spark others to experiment with these approaches and improve them.

## Appendix

Multimedia Appendix 1 Cluster quality chart for split between distressed and nondistressed users in Happify.

Multimedia Appendix 2 Heat map for intercorrelations among 200 topics and scree plot. These patterns describe largely small intercorrelations, but with a not insubstantial number of moderate, positive correlations, suggesting that factor analysis may reveal meaningful factors.Eigenvalues of the Correlation Matrix of Topic Scores.

Multimedia Appendix 3 The five topics most strongly correlated with positive emotion and life satisfaction.

Multimedia Appendix 4 All topics significantly correlated with well-being improvement and reduction. Topics predicting increased well-being.

Multimedia Appendix 5 Topics predicting increased well-being among users in the top tercile of initial well-being.

## Abbreviations

LDA: latent Dirichlet allocation
RCT: randomized controlled trial
STAGE: savor, thank, aspire, give, and empathize
