# Correction: Seeing the “Big” Picture: Big Data Methods for Exploring Relationships Between Usage, Language, and Outcome in Internet Intervention Data

**DOI:** 10.2196/jmir.8099

**Published:** 2017-12-19

**Authors:** Jordan Carpenter, Patrick Crutchley, Ran D Zilca, H Andrew Schwartz, Laura K Smith, Angela M Cobb, Acacia C Parks

**Affiliations:** ^1^ Positive Psychology Center University of Pennsylvania Philadelphia, PA United States; ^2^ Penn Medicine Social Media and Health Innovation Lab, Penn Medicine Center for Health Care Innovation University of Pennsylvania Philadelphia, PA United States; ^3^ Happify New York, NY United States; ^4^ Computer Science Stony Brook University Stony Brook, NY United States; ^5^ Department of Psychology Hiram College Hiram, OH United States

The authors of, “Seeing the 'Big' Picture: Big Data Methods for Exploring Relationships Between Usage, Language, and Outcome in Internet Intervention Data” (J Med Internet Res 2016;18(8):e241) would like to make changes to the following areas.

1. In the results section of the abstract
  - The first sentence of the results subheading in the abstract “On a measure of positive emotion, the average user improved 1.38 points per week (SE 0.01, t122,455=113.60, *P*<.001, 95% CI 1.36–1.41), about a 11% increase over 8 weeks.” **This should be replaced with:** “On a measure of positive emotion, the average user improved 1.38 points per week (SE 0.01, t122,455=113.60, *P*<.001, 95% CI 1.36–1.41), about a 27% increase over 8 weeks.”
  - The third sentence in the results subheading of the abstract “This estimate predicted that a given user would report positive emotion 1.26 points (or 1.26%) higher after a 2-week period when they used Happify daily than during a week when they didn’t use it at all.” **This should be replaced with:** “This estimate predicted that a given user would report positive emotion 1.26 points higher after a 2-week period when they used Happify daily than during a week when they didn’t use it at all.”
2. In the Study 1 Results section
  - The last sentence in the second paragraph in the Study 1 Results section “This suggests an average overall improvement of 11.04 points, or about 11%, over the course of 8 weeks.” **This should be replaced with:** “This suggests an average overall improvement of 11.04 points, or about 27%, over the course of 8 weeks.”
  - The second sentence in the fourth paragraph in the Study 1 Results section “This estimate predicted that a given user would report positive emotion that would be 1.26 points (or 1.26%) higher after a 2-week period when they used Happify daily than after a week when they didn’t use it at all.” **This should be replaced with:** “This estimate predicted that a given user would report positive emotion that would be 1.26 points higher after a 2-week period when they used Happify daily than after a week when they didn’t use it at all.”
3. In the general Discussion section
  - The last sentence in the second paragraph in the Study 1 Results section “This suggests an average overall improvement of 11.04 points, or about 11%, over the course of 8 weeks.” **This should be replaced with:** “This suggests an average overall improvement of 11.04 points, or about 27%, over the course of 8 weeks.”

While the errors do not impact the general findings of the original publication, remediation of these errors will add clarity to the interpretation of the results, as well as accurately represent the magnitude of the results.

The corrected article will appear in the online version of the paper on the JMIR website on December 19, 2017, together with the publication of this correction notice. Because this was made after submission to PubMed Central, the corrected article also has been re-submitted to PubMed Central.

