# Supplementary figures and images for: Seeing the “Big” Picture: Big Data Methods for Exploring Relationships Between Usage, Language, and Outcome in Internet Intervention Data
Source: J Med Internet Res. 2016 Aug 31;18(8):e241. doi: 10.2196/jmir.5725 (PMC5023946; doi:10.2196/jmir.5725)

## Cluster Quality

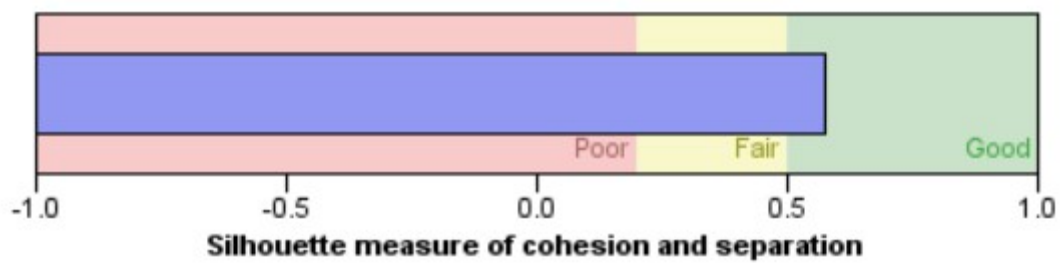

Supplement: Multimedia Appendix 1 [file jmir_v18i8e241_app1.pdf]

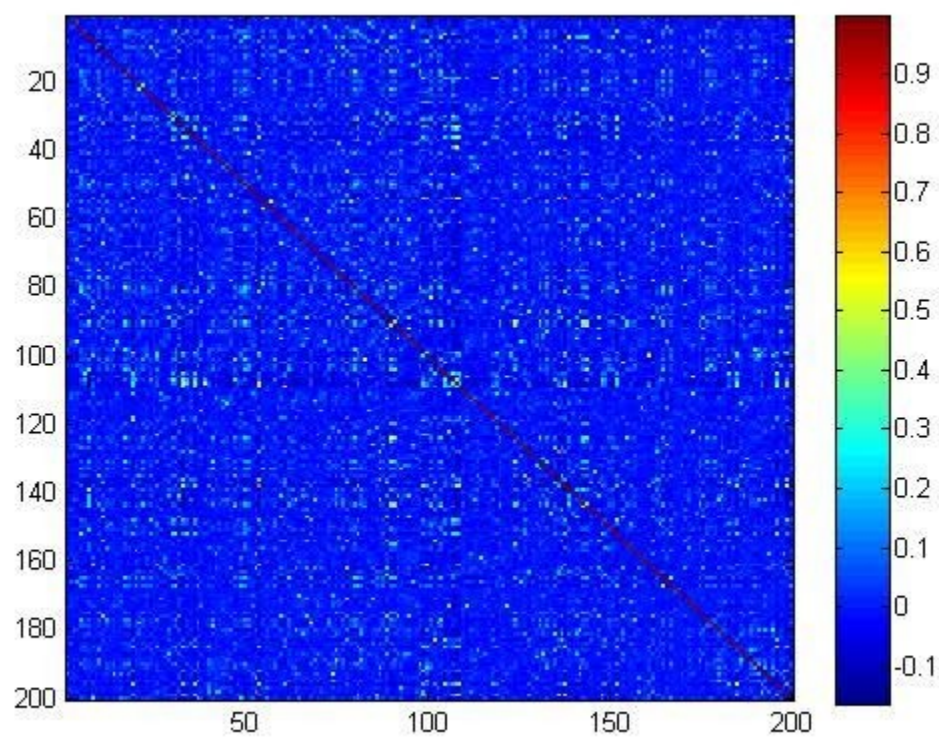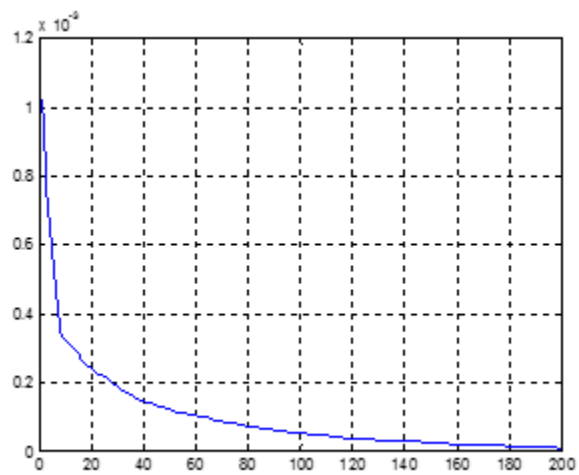

Eigenvalues of the Correlation Matrix of Topic Scores

Supplement: Multimedia Appendix 2 [file jmir_v18i8e241_app2.pdf]

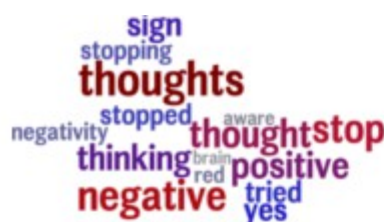

$r = .07$

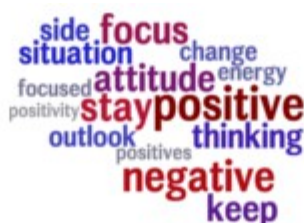

$r = .04$

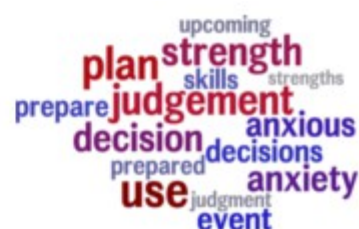

$r = .03$

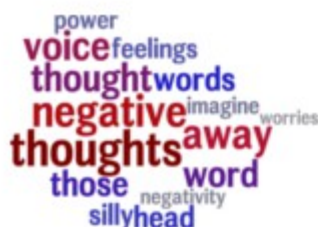

$r = .05$

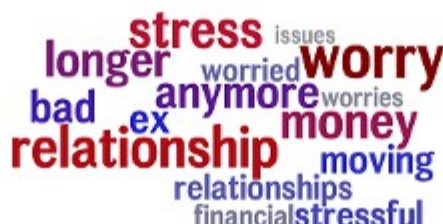

$r = .04$

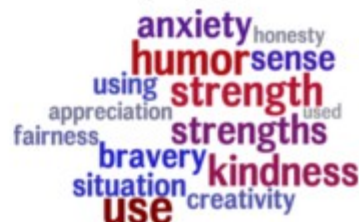

$r = .03$

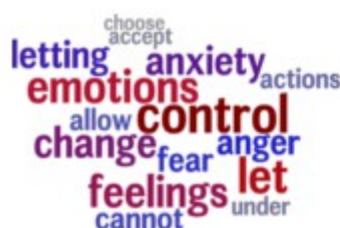

$r = .04$

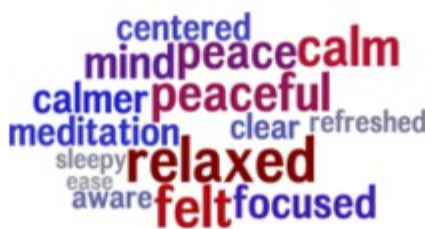

$r = .04$

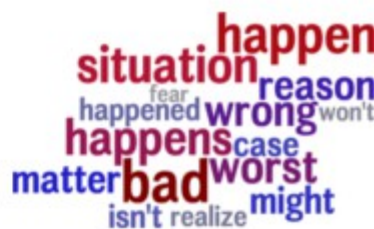

$r = .03$

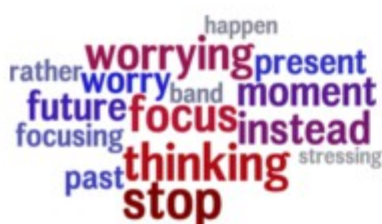

$r = .04$

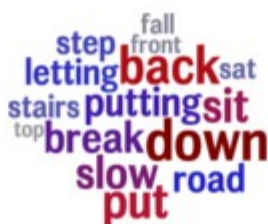

$r = .03$

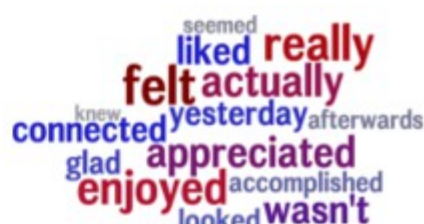

$r = .03$

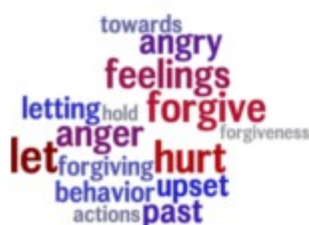

$r = .04$

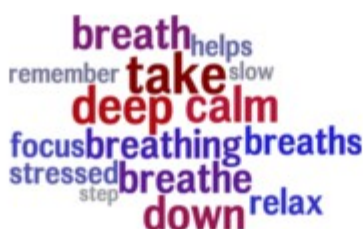

$r = .03$

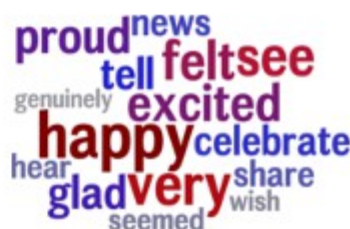

$r = .03$

Supplement: Multimedia Appendix 4 [file jmir_v18i8e241_app4.pdf]

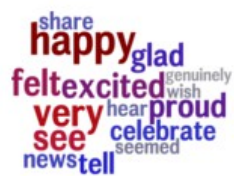

$r = .06$

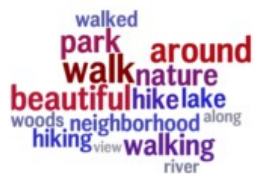

$r = .04$

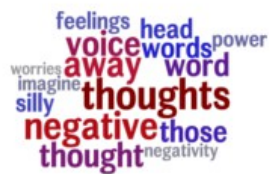

$r = .05$

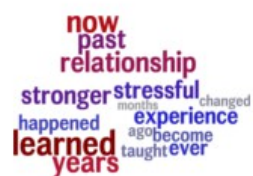

$r = .04$

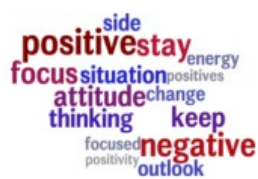

$r = .05$

Supplement: Multimedia Appendix 5 [file jmir_v18i8e241_app5.pdf]
