# Supplementary material for: Seeing the “Big” Picture: Big Data Methods for Exploring Relationships Between Usage, Language, and Outcome in Internet Intervention Data
Source: J Med Internet Res. 2016 Aug 31;18(8):e241. doi: 10.2196/jmir.5725 (PMC5023946; doi:10.2196/jmir.5725)
Supplement: Multimedia Appendix 3 [file jmir_v18i8e241_app3.pdf]

5 topics most strongly predictive of Positive Emotion

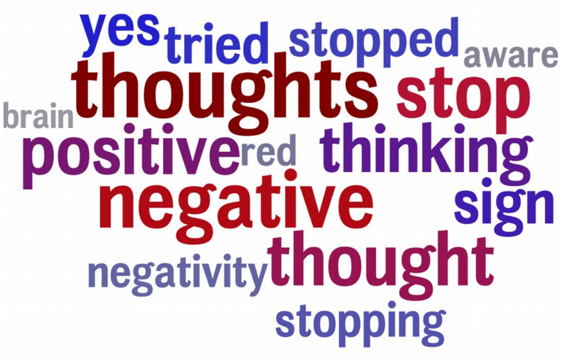

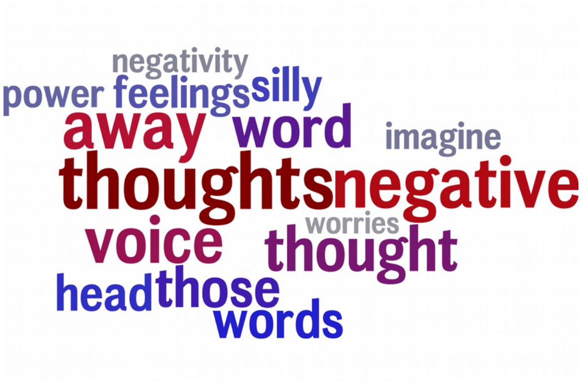

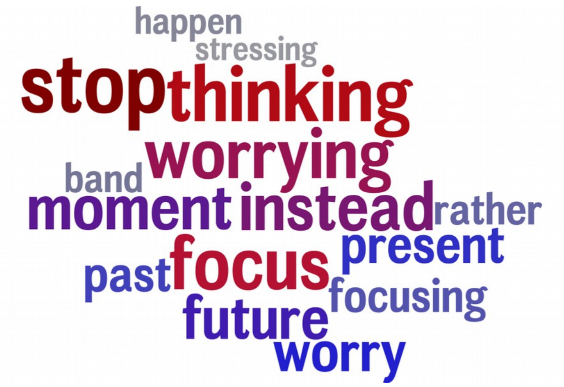

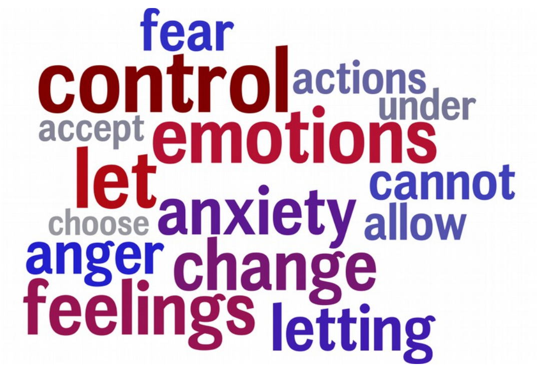

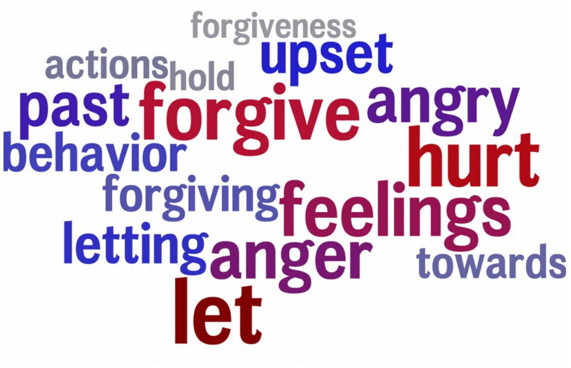

5 topics most strongly predictive of Life Satisfaction

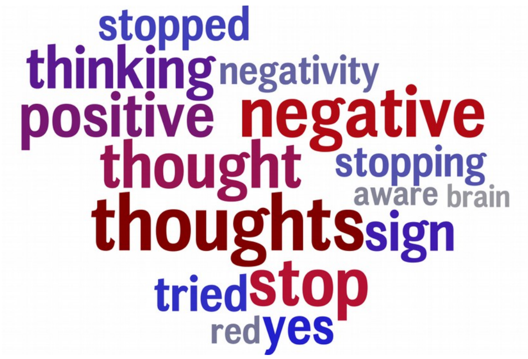
stopped
thinking
negativity
positive
negative
thought
stopping
aware
brain
thoughts
sign
tried
stop
red
yes

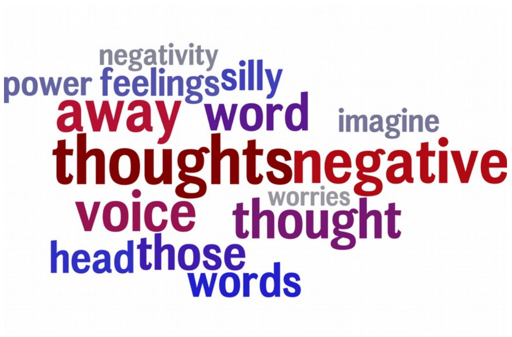
negativity
power
feelings
silly
away
word
imagine
thoughts
negative
worries
voice
thought
head
those
words

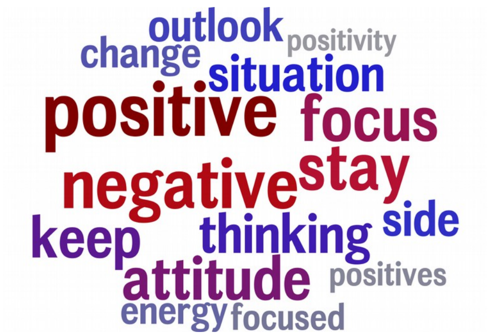
outlook
positivity
change
situation
positive
focus
negative
stay
keep
thinking
side
attitude
positives
energy
focused

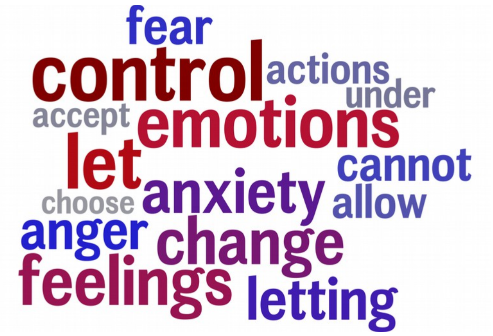
fear
control
actions
under
accept
emotions
let
cannot
choose
anxiety
allow
anger
change
feelings
letting

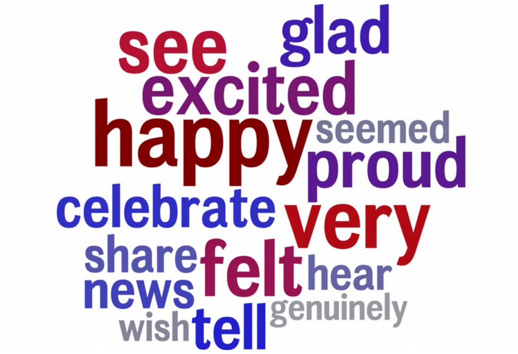
see
glad
excited
happy
seemed
proud
celebrate
very
share
felt
hear
news
genuinely
wish
tell
